# Exogenous shocks and citizens’ satisfaction with governmental policies: can empirical evidence from the 2008 financial crisis help us understand better the effects of the COVID-19 pandemic?

**DOI:** 10.1007/s11135-020-01087-2

**Published:** 2021-01-23

**Authors:** Takis Venetoklis

**Affiliations:** 1 Department of Social Research/Social Policy, University of Turku, Turku, Finland; 2 TURUN YLIOPISTO, Assistentinkatu 7, Publicum 3rd floor, 20014 Turku, Finland

**Keywords:** Individual level satisfaction, European social survey, Evaluation of governmental policies, Exogenous shocks, Global crises

## Abstract

I examine to what extend the financial crisis of 2008 affected levels of individual satisfaction with governments in general and three policy areas in particular; the economy, health services and education. I use data from the European Social Survey (9 rounds, 2002-2018, 14 countries, approx.195000 observations). Running Interrupted Time Series regressions I find that, on aggregate, there was a decrease of satisfaction with the government and the economy immediately after the crisis, but an increase for health and educational services. Longer term, satisfaction gradually increased for all the four indicators examined. In separate regressions for each country, a consistent pattern of behavior emerges. Where the short-term effect on satisfaction was negative, the long-term effect was positive, and vice versa. The switch, from short-term negative to long-term positive effect, could be attributed to the successful efforts of governments to correct the immediate adverse effects of the crisis. On the contrary, some individuals seeing the problems other countries faced, applauded their own government’s short term performance in handling the crisis. With the passing of time however, they gradually became more critical. The COVID-19 pandemic has forced governments to implement policies reviving the economy and improving services in health and the education sectors, amongst others. Results of this study may be used when measuring and evaluating the effects of the current pandemic.

## Introduction

The idea for this study matured quickly. Since the beginning of March 2020, because of the coronavirus pandemic, the university where I work, shut down completely. The same happened with schools, restaurants, churches, bars, and gyms. All gatherings with more than 10 people were forbidden. In my case, all university personnel, whether with strictly academic duties, administrative or both, were forced to work from home using all the available web resources. Literally, from one day to the next many things that were taken for granted changed. Our lives were affected, directly and indirectly, in many ways.

How did all of this begin? At the end of 2019 news agencies around the world started reporting that a new flu-type virus called COVID-19 appeared to be infecting residents at the city of Wuhan, capital of the Hubei province in China at an ever alarming rate. Although the Chinese government attempted to downplay the news, the thousands of infected cases and the death toll soon made this impossible. New infections and casualties were reported daily. In order to contain the epidemic, at the end of January 2020, the Chinese government finally took swift action and imposed a lockdown on more than 50 million people living in that city, as well as in other provinces around the country.

By that time however, the epidemic had spread in many other countries as well. Soon from Italy came news of acute health conditions, of thousands of newly infected and deaths. Iran and Spain followed. Gradually other countries, thought to be immune, reported infections and deaths as well. This was the wake-up call for many governments to start informing their citizens of the perils of this epidemic and what needed to be done in terms of hygiene and social distancing. The United States was one of the last countries to act on this situation.

The World Health Organization (WHO) albeit hesitant initially, finally classified the phenomenon a pandemic and announced harsh warnings on what would happen if measures were not implemented by all. Because of the abnormally large amount of infected individuals, the biggest fear was that health services would not cope; there would not be enough specialized equipment in intensive care units to administer adequate treatment to those in most need.

Thus, most countries imposed restrictive rules on movements of individuals and ordered lockdowns of whole sectors in the economy. Sporting events, championship series and concerts were cancelled or postponed until further notice. Naturally, the immediate effects were felt in the tourism, transportation, restaurant and entertainment sectors, and where big individual financial commitments are made such as the real estate and the car industries. Economic activity came to an abrupt stop because everything closed within a few days. That in turn caused thousands of layoffs and an upsurge in unemployment claims. Governments and central banks hurried to support the collapsing system by promising subsidies, loans with better terms and occasionally free money to those immediately affected firms and individuals alike. Work from home suddenly became the norm. Schools and universities were closed. Despite initial difficulties, teaching was soon conducted via the web. To the apprehension of many parents, the responsibility for supervising their children’s school performance fell on them.

The financial markets which had already enjoyed one of the longest bull-run in history started feeling the effects of the uncertainty caused by the havoc in everyday life. At the beginning 2020, three of the best-known indexes, the Dow Jones Industrial Average, the SandP 500 and the Nasdaq recorded all-time highs. During the second week of March all collapsed with sharp declines of more than 20%.^1^ Although all three have since rebounded, the extreme volatility has generated billions of dollars in losses to institutional as well as private investors worldwide. Oil prices fell sharply; at one point prices for the West Texas Intermediate were quoted with a minus sign.^2^.

Governments that have managed to curtail the spread of the virus better seem to enjoy popularity from their constituents whereas other governments are blamed for not taking harsher measures early enough. People started perceiving themselves as “experts” with the R_0_ indicator and the pandemic curve. They have familiarized themselves at what point then curve is estimated to reach its apex and start diminishing. The dilemma of social distancing and personal hygiene versus herd immunity became the forefront much debate. Many countries grappled with the questions of how long whole areas or even whole countries can remain under lockdown without generating irrevocable damage to the economy.

How much liquidity is needed to be injected into the economy so that people and enterprises survive the first shock? For how long can this support continue? Should central banks reduce the reserve requirement ratios for commercial banks, should they reduce discount rates, should they buy bonds held by commercial banks or should they even print new money^3^? Must countries with exceedingly high debt to GDP ratios nonetheless attempt to borrow from the financial markets to revive their economies?^4^

The COVID-19 pandemic is still an ongoing event that no one knows how it will actually develop in the future. The second wave observed during the autumn of 2020 struck with greater intensity than many had estimated. Some acknowledge that a third wave might be on its way in the spring of 2021. Regardless of the virus’s potency, however, its repercussions on billions of people will be felt for years to come. The sudden and extreme measures imposed by governments to fight the pandemic, combined with the fear of contracting the lethal virus itself, have generated considerable anxiety and mental illness amongst the general population (Fofana et al. 2020). Vaccines to immunize against the virus have been produced and have recently received licensing. Nonetheless, even if these vaccinations are implemented in sufficient numbers for the general population, it will take a considerable amount of time for socioeconomic conditions to return to pre-virus period levels. It is thus interesting from a sociological perspective to investigate how individuals reacted in the short- and long-term, during another calamity of the recent past.

The financial crisis of 2008 was not health-related. It was the subprime problem in the US that burst, bankrupted Lehman Brothers and created a tsunami of financial collapses initially and fiscal problems in many countries later (Ervasti et al., 2019, p. 1210). The two crises are indeed not completely comparable. A significant difference is the mindset of the people affected by both. In 2008 many were suddenly faced with a financial collapse and the uncertainty of the potential unemployment ahead. With the COVID-19 pandemic the negative financial repercussions are only one aspect. The restrictions on movement due to lockdowns and, as discussed above, the fear of contracting the lethal virus have created even harsher living conditions for millions. And yet, the two crises are similar at a more macro level. They were both sudden crises, were generated by exogenous events and they effected directly or indirectly all countries around the globe.^5^

In examining the 2008 crisis we have the opportunity to investigate how different countries coped with the sudden exogenous shock, both short and long term. In particular, we can examine the opinions of citizens about their governments and how they evaluate the policies on the economy, education and health services. This might provide a gauge in the future for comparing it to the current COVID-19 crisis. The European Social Survey (ESS) has the suitable data for this. Respondents are asked how satisfied they are with their government, the economy, education and the health services. All these four indicators provide a reasonable assessment of how citizens evaluate their government. Although the majority of the literature is focusing on the level of trust citizens have in political and impartial institutions, examining the satisfaction level with respect to specific policies is a more direct and practical approach in measuring the success or failure of government activities concretely.

### A few theoretical considerations. Why measure the satisfaction of citizens?

Morgeson (2014, p. 7) asserts that the satisfaction of citizens refers to the “…individual citizens' (in the aggregate) happiness or contentment (or what another author called “fulfillment response”) with an experience or experiences with the services (or goods, or processes, or programs) provided by the government bureaucracies and administrative institutions.” Surveys measuring satisfaction of citizens with governments in general but also with different policies in particular, have been conducted since the 1970s. They have been popular especially with regard to services provided at local and municipal level (Stipak 1979). Such surveys have been conducted with increasing frequency in recent years (Dutil et al. 2010, p.31; Howard 2010, p. 66).

The basic logic behind these feedback mechanisms stems from the hypothesis that in a democratic institutional framework governments must be responsive to the preferences of their constituents regarding public policies. As Rosset et al. (2017, p. 796) note, the “democratic rule” implies that there is a connection between the citizens and the state and that citizens’ preferences are considered by the political institutions that govern the country. Huber and Powell (1994, p. 293) even assert that what constituents prefer, is best represented by the preferences of the median voter. This feedback approach also has a theoretical framework similar to that discussed in Campbell (2012); it resembles the responsive approach due to the democratic obligations of elected governments. It says that policies obviously influence citizens’ behavior, which then gives feedback (sometimes “feed forward” to describe the temporal ordering of things) and thus influences future policy formulation and implementation (Ziller 2019, p. 287).

In classifying this feedback mechanism under an even larger theoretical framework we may need to go back to the 1980s in New Zealand and the early 1990s in the UK, when the first ideas regarding the New Public Management (NPM) paradigm appeared (Hood 1991, cited in McLaughlin and Osborne 2002, p.1). The basic goal of the NPM is to increase and improve the efficiency and the effectiveness of services provided by the public sector. According to Ariely (2011, p.999) the premise guiding these changes is obvious: better public institutional performance leads to satisfied citizens, which in turn generates positive evaluations of governments as a whole. The emphasis on performance reflects among others the ever-growing problem of fiscal austerity that must be implemented due to the economic deficits that most governments face (Van Ryzin 2015, p. 426).

In measuring citizens’ satisfaction, the other major theoretical approach is based on the assumption that planners and implementers of policies need “evidence” when making key decisions (Nutley et al. 2007). Dutil et al (2010, p. 31) elaborate on this further and at the same time justify the use of satisfaction surveys as follows: “Proponents of evidence-based decision-making persuasively argue that managers require reliable and impartial data on policy and administrative problems, to effectively navigate the complexities and constraints of the contemporary policy environment. Citizens satisfaction surveys are thus attractive, because they promise simultaneously to enhance public input into government and the methodological rigor of the evidence used in official decision making”.

The feedback process is not geared solely towards the government’s performance, in general. There are instances where feedback focuses on specific policies and measures. For example citizens are concerned about education and health care policies, among others (Stecker and Tausendpfund 2016, p. 496). Moreso, voters are eager to evaluate their governments’ economic performance because depending on its success, it can affect directly their wellbeing. The evidence on the link between economic performance and citizens’ satisfaction goes back many decades and remains topical. (see for example, Fiorina (1978), Healy and Malhotra (2013), Dassonneville and Lewis-Beck (2019), Gahner-Larsen et al. (2019)).

Furthermore, satisfaction with government policies is closely correlated with another very popular theme in social and political sciences, trust. Most authors would agree that satisfaction is a prerequisite for trust. Van de Walle and Bouchaert (2003, p. 892) state that many accept the implicit assumption that “…better performing public services will lead to increased satisfaction among their users, and this, in turn, will lead to more trust in government.” This has shown to be true both regarding trust with the government in general, but also with specific policies in particular. For example Van Ryzin et al (2004, p.332) have used the American Customer Satisfaction Index (ACSI) model to capture the process through which residents of New York evaluate the different services provided by the city. They clearly show that satisfaction with the government comes temporarily before trust (ibid, p. 333, Fig. 2). Within his own theoretical model, Vigoda-Gadot (2007, p. 290, Fig. 1) depicts a similar relationship. Finally, satisfaction with particular services such as health care is also mentioned as a prerequisite for trust in the government (Christensen and Laegreid 2005).

### Goals and hypotheses

The main goal of the study is to examine and compare the potential impact of the 2008 financial crisis on the individual levels of satisfaction in different European countries. I hypothesize that, on average, the 2008 crisis has contributed in diminishing the levels of satisfaction short-term and perhaps long-term as well. The analysis is thus based on these 2 chronological horizons. In both cases, I first attempt to measure potential associations between levels of satisfaction and the financial crisis. I then rank the intensity of the relationship - if any - by country and evaluate the result as positive, negative or non-significant.

The paper proceeds as follows. Below I describe the data and the variables based on which I conduct the empirical analysis. In the methods section, I discuss certain theoretical consideration regarding the regression models applied. Then, I commend on the reported results. In the final section, I summarize and discuss the findings which I also link to the current COVID-19 crisis. In addition, I list several caveats, briefly mention other interpretations that could also be used in explaining the empirical findings and propose certain areas for future research.

## Data

My main goal is to measure dynamically the short-as well as the long-term effects of the crisis. For this I use data from 14 countries and responses collected in all 9 rounds of the ESS.^6^ Of the 38 countries that at some point in time were surveyed, only those 14 participated in every round (2002-2018).

### Dependent variables

For the analysis I use four dependent variables. One measures the overall satisfaction of the respondents with the activities of the government in general and three measure the level of satisfaction with particular government policies: the economy, health services and education. All their values ranged from 0 to 10. The exact wording of the ESS questions based on which the responses were recorded were as follows:

How satisfied are you with

- The national government
- The recent state of economy in country
- The state of health services in country nowadays
- The state of education in country nowadays

### Independent variable of interest

The independent variable of interest is the ESS round. With 9 different surveys conducted between 2002 and 2018, I treat the round in the models as continuous with values ranging from 1 to 9. I run several Interrupted Times Series (ITS) regressions (see below). With an ITS specification, I can estimate the rate of growth of satisfaction (its slope) throughout the period under scrutiny, both before and after the crisis and determine the long term effects of the crisis, if any. In addition, I can compare the levels of satisfaction between in years 2008 and 2010 (rounds 4 and 5), thus measure the immediate effects of the 2008 crisis on satisfaction.

### Control variables

People’s evaluations of governments measured through levels of satisfaction and trust are found to differ based on age, gender or socioeconomic background (see e.g. Lyons et al. 1992; Van Ryzin et al. 2004; Christensen and Laegreid 2005; Van de Walle 2007; Van Ryzin 2015). Van Ryzin (ibid, p.435) in particular, notes that education “…helps control the differences across respondents in the knowledge of and experience with (local) government”.

The correlation between satisfaction and trust in the government is positive and in general rather high. For instance, Vigoda-Gadot (2007, p.297, Table 2) reports a figure of 0.67. Thus, the same controls have been used not only in models measuring satisfaction but trust as well, where research is even more extensive. (see, for example Brewer et al.( 2004, p.102), Fridberg and Kangas (2008, pp.79–82), Arpino and Obydenkova ( 2020, p.409, Table 3), Torrente et. al. (2019, pp.646–647), and Daskalopoulou (2019, pp.290–291)). Specifically, Rudolph and Evans (2005, p.661) find that at the individual level, political trust matters more to conservatives than to liberals. Jost et al. (2009) also find that political ideology is a factor that is accepted as important when measuring people’s attitudes towards officials who govern. Also Haugsgjerd (2018, p.628) uses household income, educational background, employment status, age and gender as controls in his regressions measuring trust. Finally Currie et al. (2015) investigated how the great recession affected mothers’ health. They found that mothers with less education, unmarried and from minorities experienced a greater deterioration in their health compared to those who were white, married and had gone to college.

Following the aforementioned empirical research, in my models I use the following individual-level control variables: Gender, (level of) Religiosity, Age, Age squared, Happiness Level, Social Activity, Political Orientation, Years of Education, (level of) Subjective Health, Subjective Financial Status and Country (Dummy). (Table 1).

**Table 1:** List of variables

Variable	N	Mean	Std. Dev	Min	Max
Satisfaction with the					
National government (GOV)	221,790	4.3390	2.4095	0	10
Recent state of economy (ECO)	223,913	4.7628	2.4573	0	10
State of health services (HLT)	226,510	5.5974	2.4527	0	10
State of education (EDU)	218,300	5.6210	2.2686	0	10
Round	228,141	4.8561	2.5659	1	9
Gender	227,975	1.5260	0.4993	1	2
Religiosity	226,602	4.5372	3.0199	0	10
Age	228,014	49.431	18.002	18	99
Subjective happiness level	227,308	7.4518	1.8447	0	10
Social activity	227,657	4.9822	1.5482	1	7
Political orientation (left/right axis)	205,895	5.0476	2.1565	0	10
Years of education	228,014	12.434	4.2332	0	30
Subjective health status	227,870	2.2390	0.9018	1	5
Coping with own finances	223,618	1.9096	0.8129	1	4
Country	228,014	7.1430	3.9087	1	14

## Methods

Policy impacts are best measured by conducting so called social experiments. The focus is on the population of interest or a sample of its units, which is randomly divided into 2 groups: one that is exposed to the policy (the treatment) of interest and one that is not. The latter group is often used to depict the counterfactual situation—what would have happened to the policy indicator of interest had the experimental group not been exposed to the treatment. The random assignment to the two groups ensures that whatever different characteristics exist between them cancel out and the differences that remain on the indicator is the impact of the policy intervention.

In standard notation,

- U = Universe of all units of interest.
- u = One unit in that universe.
- Y(u) = An impact related to each u.
- T = Causes of Y(u). They can be
- i = treatment/intervention (policy, program, measure, condition of interest, exogenous event).
- c = control (any other treatment or no treatment–the most usual).

The effect of the intervention on the units of interest is always based on a relative comparison. It is the average difference of the impact on the units given (“|”) they were treated, less the impact on the units given they were not treated, or 

$${Y} (u)|{T} = {i} - {Y}(u)|{T} = {c}$$

As discussed, through randomization, we attempt to make the two groups as identical as possible, because we cannot observe simultaneously the same units under 2 different treatment regimes. For a non-experimental ex-post evaluation- as it is the case here - the best case scenario is having 2 populations, with measurements on the indicator of interest; one that is exposed to the treatment and the other that is not exposed. Preferably the observations cover the periods before and after the intervention, for some time. However since the classification is not done at random, it becomes problematic because we do not have two groups of data that differ by just that exposure to the intervention, on average. This, in turn, implies that their measured differences in the indicator might not be solely due to this intervention (Holland 1986; Imbens and Wooldridge 2009).

An even weaker scenario is where the intervention covers all the available population. As I show later, this compels us to approximate the counterfactual population using a very weak assumption on its behavior; that it will continue to behave the same post-treatment, as during pre-treatment. This in fact is the main methodological constraint with the current data and the research questions. On the other hand, measurements on the indicators of interest are present, both pre-and post-intervention. Hence, we can perhaps identify trends in growth, positive or negative, before and after 2008, the seminal year during which the crisis erupted.

In brief, we may think of the financial crisis as the policy treatment. By definition the crisis is an exogenous event that produces a global shock, thus covers all the population of the countries examined. The objective is to establish whether the crisis has had an impact on the levels of satisfaction of the people surveyed. In my analysis I use an Ordinary Least Squares (OLS) fixed effects^7^ regression specification called Interrupted Time Series.

### Considerations when comparing two periods

Here I discuss what should be considered when measuring the levels of individual trust between two periods. I compare how the four types of satisfaction grew between 2008 and 2010. Following the classification suggested by Langbein and Felbinger (2006, p.118) the data structure I utilize is the Pretest–Posttest (Single-Group) Design.^8^

$${Satisfaction}^{C}_{t-}\, {Crisis}_{t-{\it{2008}}}\, {Satisfaction}^{T}_{t{+}}\, {,\, where}$$

   Satisfaction = Level of satisfaction (Policy Impact; our dependent variable of interest).

      Crisis = Financial crisis (Policy Intervention-treatment, the exogenous event).

      T = (T)reated group of individuals.

      C = Untreated group of individuals–The (C)ontrols.

      t-= time just before the crisis occurred (better, time before the crisis was felt), in 2008.

      t = time when the financial crisis occurred, in 2008.

      t +  = time after financial crisis, 2010.

I utilize this design because the program is applied horizontally. That is, all units are potentially exposed to the treatment. In our case, the treatment is the financial crisis which has potentially affected all individuals in the countries surveyed. The impact of the financial crisis on satisfaction is measured as the difference in the mean levels of satisfaction of individuals before and after the crisis, controlling for certain variables. The ESS survey conducted in each country is using a random representative sample of individuals during each round. In other words the individuals interviewed in one round are not the same ones the round that follows. Thus we are not able to reduce our estimates’ bias by eliminating all fixed individual characteristics, which could be achieved when analysing panel data. The other weakness in the design is that the surveys in these two rounds occur at different times. Hence all exogenous factors during one period that might influence ones’ behaviour are different the next. Therefore it is natural to expect a “temporal heterogeneity” of conditions, and consequently of behaviour. The difference of the 2 rounds calculation performed here assumes nonetheless a “temporal homogeneity” amongst the individuals surveyed, which is a weak assumption as discussed earlier. On the other hand, the random selection of the respondents in each round helps perhaps to cancel out the potential differences in individual characteristics, on average.

### Longer term analysis

In contrast to the previous short term analysis, here I analyze more than 2 periods of collected responses concurrently; four before and five after the financial crisis. Again, following the classifications by Langbein and Felbinger (2006, p.120) and Shadish et al. (2002, p.175),^9^ the data structure I examine is once more of the “One group before-after design” type with the element of an Interrupted Time-Series added to it. It is depicted as follows:

$$\it \it \text{ Satisf } ^ \text{ C } _{{ 2002 }} \text{ Satisf } ^ \text{ C } _{{ 2004 }} \text{ Satisf } ^ \text{ C } _{{ 2006 }} \text{ Satisf } ^{ C } _{{ 2008 - }} \text{ Crisis } ^{ C } _{{ 2008 }}\text{ Satisf } ^ \text{ T } _{{ 2010 }} \text{ Satisf } ^ \text{ T } _{{ 2012 }} \text{ Satisf } ^ \text{ T } _{{ 2014 }} \text{ Satisf } ^ \text{ T } _{{ 2016 }} \text{ Satisf } ^ \text{ T} _{{ 2018 }}$$

As discussed previously, the treatment (the financial crisis) is applied horizontally. All individuals surveyed after 2008 were potentially exposed to the consequences of the financial crisis. This again assumes temporal homogeneity meaning that, in the absence of the crisis, the development of satisfaction levels would have continued to evolve as in the pre-crisis period, linearly.^10^ As there are now observations in more than two time periods, both before and after the intervention, this design can reveal changes in the growth of satisfaction that could perhaps be attributed to the financial crisis. It measures dynamically how satisfaction preferences evolve throughout the period under scrutiny and how the financial crisis may have contributed to this growth, be it positive or negative.

### Interrupted time series models

One method, of establishing perhaps a causal relationship between the 2008 crisis and the levels of satisfaction, is by comparing growth trends of satisfaction before and after the crisis. In cases where we have a treatment covering the entire population at hand, we estimate the slopes of the dependent variable of interest before and after the intervention and compare the two. In a nutshell, if the difference is statistically significant with a plus sign, this indicates a positive effect; if the sign is negative, it indicates the opposite.^11^

I follow Wagner et al. (2002, p. 301) and Lopez-Bernal et al. (2017) and apply an Interrupted Time Series regression or segmented regression model. Penfold and Zhang (2013, p.S38) assert that analysis based on ITS “…is arguably the strongest quasi-experimental research design”. Zhang et al. (2009, p. 143) concur “ITS designs, especially when they involve analysis of comparison series, are the strongest observational designs to evaluate changes caused by interventions because they can account for the pre-intervention level and trend of the outcome measures". A limitation of the method is that it requires a minimum of 8 time observational instances before and after the intervention (Penfold and Zhang, 2013, p.S43). I only have about half such instances in my data (4 rounds before and 5 rounds after). Although estimations can be calculated, they result in coefficient confidence intervals that are too narrow, consequently exaggerating precision. In such cases it is recommended that one runs the models using repeated samples via bootstrapping (Efron and Tibshirani 1993; cited in Zhang et al. 2009, p.146).^12^ Abiding by the standard rules of ITS analysis, the following segmented regression model is used:

$${b} _{\it{0}}\,+\, {b}_{\it{1}} {T}\,+\,{b}_{\it{2}} {D}\,+\, {b}_{\it{3}} {P}\,+\, {controls\, +\, error,}\, {where}$$

Y depicts levels of satisfaction.

T is the time variable (here the round). It is continuous with values ranging from 1 to 9. Its b_1_ coefficient is interpreted as the slope of satisfaction for the period before the crisis (rounds 1–4).

D is a dummy variable depicting the two periods before and after the crisis with values.

0 if the round is one of 1–4 (2002–2008), 1 if the round is of one of 5–9 (2010-2018). Its coefficient b_2_ denotes the change (difference) in mean value of satisfaction immediately after the crisis; that is, between round 4 (2008) and round 5 (2010).

P is another time variable. It represents the period following the 2008 crisis. The coefficient b_3_ denotes the difference in slope (growth) after the crisis versus the slope before. To find the slope for the period after the crisis we simply add b_1_ + b_3_. Notwithstanding the limitations of the evaluation design discussed earlier, this impact indicator takes under consideration the growth trends of satisfaction dynamically.

## Results

### Models using all country data together

Table 2 shows the relevant ITS estimations for each of the four dependent variables using the individual controls of Table 1. Note that the number of observations is considerably less than those depicted in Table 1 due to list-wise deletions.

**Table 2:** Aggregate models (all countries)

Model	1	2	3	4
Satisfaction with:	Government	Economy	Health services	Education
Male	Ref.	Ref.	Ref.	Ref.
Female	−0.0825 ***	−0.2647 ***	−0.3439 ***	−0.1115 ***
Religiosity	0.0819 ***	0.0519 ***	0.0551 ***	0.0578 ***
Age	−0.0320 ***	−0.0217 ***	−0.0513 ***	−0.0267 ***
Age X Age	0.0004 ***	0.0002 ***	0.0006 ***	0.0002 ***
Subjective Happiness level	0.1578 ***	0.2111 ***	0.1606 ***	0.1527 ***
Social meetings	−0.0209 ***	−0.0150 ***	0.0145 ***	0.0019
Political orientation	0.0925 ***	0.0639 ***	0.0314 ***	0.0230 ***
Years of education	0.0096 ***	0.0118 ***	−0.0057 ***	−0.0365 ***
Subjective Health status: 1	Ref.	Ref.	Ref.	Ref.
Subj. Health status: 2	−0.1296 ***	−0.1296 ***	−0.1490 ***	−0.0975 ***
Subj. Health status: 3	−0.2748 ***	−0.3011 ***	−0.2808 ***	−0.1887 ***
Subj. Health status: 4	−0.4061 ***	−0.4477 ***	−0.3880 ***	−0.2405 ***
Subj. Health status: 5	−0.4772 ***	−0.4542 ***	−0.6572 ***	−0.3667 ***
Cope with own finances: 1	Ref.	Ref.	Ref.	Ref.
Cope with own finances: 2	−0.2807 ***	−0.4682 ***	−0.2120 ***	−0.0906 ***
Cope with own finances: 3	−0.5971 ***	−0.9343 ***	−0.2894 ***	−0.1969 ***
Cope with own finances: 4	−0.8087 ***	−1.3243 ***	−0.3554 ***	−0.2118 ***
Country: BE	Ref.	Ref.	Ref.	Ref.
CH	1.2096 ***	1.0101 ***	−0.7023 ***	−0.0288
DE	−0.3291 ***	0.1243 ***	−2.0099 ***	−1.5427 ***
ES	−0.8385 ***	−1.1732 ***	−1.6080 ***	−1.5683 ***
FI	0.7657 ***	0.8097 ***	−0.5105 ***	1.3182 ***
FR	−0.7922 ***	−1.4589 ***	−1.0546 ***	−1.3923 ***
GB	−0.4186 ***	−0.5365 ***	−1.6102 ***	−0.7783 ***
HU	−0.3835 ***	−0.8711 ***	−3.2412 ***	−1.4132 ***
NL	0.4148 ***	0.4801 ***	−1.2870 ***	−0.4877 ***
NO	0.3576 ***	1.7012 ***	−1.1633 ***	0.1530 ***
PL	−1.0813 ***	−0.6756 ***	−3.5630 ***	−1.0409 ***
PT	−0.9045 ***	−1.4615 ***	−2.9233 ***	−2.0882 ***
SE	0.5117 ***	0.5429 ***	−1.6474 ***	−1.0094 ***
SI	−0.7226 ***	−0.9999 ***	−2.2860 ***	−1.0753 ***
(T) b1: slope 02-08	0.0280 ***	0.0174 *	0.1540 ***	0.0661 ***
(D) b2: diff 08-10	−0.3295 ***	−1.1530 ***	0.7080 ***	0.2491 ***
% change b2	−7.1456	−20.8933	13.4392	4.4893
(P) b3: diff in slopes (10-18)–(02−08)	0.0367 ***	0.2040 ***	−0.1361 ***	−0.0361 ***
b1+b3: slope 10–18	0.0647	0.2214	0.0179	0.0300
Constant	3.4780 ***	3.7054 ***	6.5282 ***	5.9860 ***
N	197259	198307	199590	193916
R-squared	.1726	.3104	.2418	.2134
aic	865387	839978	863567	817222
bic	865713	840304	863894	817548

Asterisks denotes statistical significance levels at *p*-values *p < 0.05, **p < 0.01 and ***p < 0.001

The individual controls are statistically significant in almost all cases, ceteris paribus. Compared to women, men seem to be on average more satisfied with all four indicators under scrutiny. The more religious activity you pursue the more satisfied you are with the government and with its 3 policy areas. The same positive correlation is found with satisfaction and political orientation. The more conservative you are in the left–right political axis, the more positive you evaluate government’s policies but also the government in general. Age has a curvilinear relationship with satisfaction. At a younger age you tend to be more critical, but as you grow older your opinions on how governments perform become perhaps more pragmatic and realistic. The happier you are with your own life in general the more satisfied you are with the government and its policies. The opposite is reported when it comes to health. The worse subjective health level you report, the less satisfied you are. The same applies to personal finances. The more difficulties you have making ends meet, the less satisfied you are with the ones who govern you and the policies they implement.

In 2 individual controls the results are not as uniform however. Social activities correlate negatively with government satisfaction, negatively with economic policies, positively with health and seem to have no associations with education in a statistically significant way. In addition, those that are highly educated have a critical view on health services and education, but are satisfied with the government in general and with its economic policies.

### Coefficients of the independent variables of interest T, D and P

To reiterate, in the absence of the crisis, the basic assumption is that satisfaction would have continued to grow between 2010 and 2018 (after the crisis) at the same pace as during the pre-crisis period, that is, between 2002 and 2008. Lopez-Bernal et al. (2017, pp.350–351, Fig. 2) present six different ITS impact models based on the immediate level of the dependent variable just after an intervention, as well as its slopes before and after the intervention.^13^ As discussed above, b_1_ depicts the growth of satisfaction from 2002 up until before the crisis. In all four models it comes out statistically significant and is gradually increasing across all 14 countries in our database, on average. However, the effects after the crisis are mixed. Immediately after, the satisfaction with the government and with its economic policies-depicted by b_2 _dropped; it increased for policies regarding health and education. Thereafter the situation again reversed somewhat. The growth in satisfaction (b_3_) after the crisis both regarding the government in general and with its economic policies - was on average higher during the period of 2010–2018 compared to 2002–2008. For health and education policies satisfaction still grew, but at a lower pace compared to the pre-crisis rate.

### Individual country analysis

Although the previous analysis provides on aggregate some insight on the potential effects of the 2008 crisis on satisfaction in these 14 countries, we still do not have a clearer picture of the dynamic process that has taken place within each country. Note that the country dummy coefficients do not always have the same sign across the 4 models and their mean satisfaction differences come out statistically significant.

Following Venetoklis (2019, pp. 3042–3043), for each of the four dependent variables of interest, I run 14 separate Interrupted Time Series OLS regressions. With these I depict the slope (growth rate) before the crisis, the change -if any - in the levels of satisfaction during the two-year period immediately after the crisis and the difference in slopes (growth rates) between the period before and after the crisis. In essence, I interact the country dummy with the control variables in each of the Models 1–4. Tables 3, 4, 5 and 6 show the results of the models for each dependent variable of interest (satisfaction with government, the economy, health services and education). Each table contains 14 models, one for each country examined. I list the 3 coefficients of interest, b_1_, b_2_ and b_3_ with their statistical significance. For b_2_ I also calculate the respective elasticity, that is, how much satisfaction changed percentage wise from 2008 to 2010.^14^

**Table 3:** Satisfaction with government per country

Model	Country	b1: slope 02 – 08	b2: diff 08 – 10	b2: % change 08 – 10	b3: diff in slopes (10 – 18) –(02 – 08)	b1 + b3: slope 10 −18	N	R-squared	aic	bic
5	BE	-0.3416 ***	-2.1860 ***	-37.3710	0.5073 ***	0.1657	14,207	0.0775	60,945	61,088
6	CH	0.1943 ***	0.2237	3.7914	-0.0434	0.1509	13,336	0.1060	53,752	53,894
7	DE	0.3407 ***	0.8626 ***	23.5495	-0.2267 ***	0.1140	22,376	0.1429	96,260	96,412
8	ES	-0.1229 ***	-2.8836 ***	-54.8671	0.2649 ***	0.1420	13,486	0.1481	60,593	60,736
9	FI	0.0303	0.5469 ***	10.5267	-0.2365 ***	-0.2062	16,238	0.2086	66,690	66,836
10	FR	-0.2020 ***	-0.8958 ***	-21.6886	0.1317 ***	-0.0703	15,247	0.1037	66,026	66,171
11	GB	-0.2216 ***	0.1738	4.2735	0.1471 ***	-0.0745	16,710	0.1041	73,903	74,050
12	HU	-0.9669 ***	-2.0475 ***	-41.1154	1.0468 ***	0.0799	11,410	0.1736	52,516	52,655
13	NL	0.4780 ***	0.8753 ***	18.7790	-0.3816 ***	0.0964	15,196	0.1579	60,283	60,428
14	NO	0.2510 ***	1.2606 ***	28.5688	-0.2179 ***	0.0331	13,577	0.1102	57,230	57,373
15	PL	0.0787 **	-0.4072 *	-10.9870	0.0898 **	0.1685	11,863	0.0971	53,581	53,721
16	PT	0.1329 ***	-3.8525 ***	-71.3306	0.4883 ***	0.6212	11,171	0.1377	49,354	49,493
17	SE	-0.016	2.5335 ***	65.4183	-0.3030 ***	-0.319	13,995	0.1140	59,989	60,133
18	SI	0.0124	-3.3065 ***	-59.5389	0.2721 ***	0.2845	8447	0.1508	37,767	37,900

Asterisks denotes statistical significance levels at *p*-values *p < 0.05, **p < 0.01 and ***p < 0.001

**Table 4:** Satisfaction with economy per country

Model	Country	b1: slope 02 –08	b2: diff 08 – 10	b2: % change 08 –10	b3: diff in slopes (10 – 18) – (02 –08)	b1 + b3: slope 10 – 18	N	R-squared	aic	bic
19	BE	-0.2452 ***	-1.5094 ***	-25.4729	0.3885 ***	0.1433	14,236	0.1425	58,221	58,365
20	CH	0.4003 ***	1.3098 ***	23.1195	-0.2581 ***	0.1422	13,523	0.2298	54,053	54,196
21	DE	0.4469 ***	1.2604 ***	28.9229	-0.1611 ***	0.2858	22,579	0.3576	95,673	95,826
22	ES	-0.4260 ***	-5.5911 ***	-80.6921	0.8459 ***	0.4199	13,614	0.2512	57,406	57,548
23	FI	-0.0086	-0.4203 ***	-6.7771	-0.0334	-0.042	16,281	0.1772	64,351	64,497
24	FR	-0.2649 ***	-1.6333 ***	-37.8081	0.3560 ***	0.0911	15,233	0.1040	62,855	63,000
25	GB	-0.5595 ***	-4.2390 ***	-62.6093	0.9148 ***	0.3553	16,644	0.1795	70,620	70,767
26	HU	-0.7349 ***	-3.8116 ***	-65.8654	1.1527 ***	0.4178	11,536	0.2357	48,717	48,856
27	NL	0.1544 ***	-1.5374 ***	-23.6134	0.1578 ***	0.3122	15,256	0.1671	58,730	58,875
28	NO	0.2040 ***	2.4597 ***	43.2065	-0.3453 ***	-0.1413	13,593	0.1675	56,350	56,493
29	PL	0.4860 ***	-0.1135	-2.6793	-0.1872 ***	0.2988	11,830	0.2217	49,745	49,885
30	PT	0.0621 *	-3.0275 ***	-64.7193	0.4311 ***	0.4932	11,194	0.1437	45,888	46,027
31	SE	0.0557 *	1.2274 ***	23.8923	-0.0852 **	-0.0295	14,190	0.1793	58,260	58,404
32	SI	0.0509	-4.8656 ***	-73.5797	0.5151 ***	0.566	8598	0.2434	36,602	36,736

Asterisks denotes statistical significance levels at *p*-values *p < 0.05, **p < 0.01 and ***p < 0.001

**Table 5:** Satisfaction with health services per country

Model	Country	b1: slope 02 –08	b2: diff 08– 10	b2: % change 08 – 10	b3: diff in slopes (10 – 18) –(02 – 08)	b1 + b3: slope 10 –18	N	R - squared	aic	bic
33	BE	0.1696 ***	1.2257 ***	18.4833	-0.2513 ***	-0.0817	14,328	0.0874	53,639	53,783
34	CH	0.2587 ***	0.8701 ***	13.7566	-0.2064 ***	0.0523	13,602	0.0645	57,755	57,898
35	DE	-0.0699 **	-1.0086 ***	-17.4783	0.3280 ***	0.2581	22,672	0.1283	101,230	101,383
36	ES	0.2213 ***	0.8192 ***	15.8213	-0.2930 ***	-0.0717	13,634	0.0719	60,961	61,103
37	FI	0.0227	-0.3603 **	-5.0945	0.0742 **	0.0969	16,365	0.0797	66,143	66,289
38	FR	0.1278 ***	0.5504 ***	9.4553	-0.1253 ***	0.0025	15,312	0.0868	65,539	65,684
39	GB	0.3056 ***	2.7380 ***	64.9598	-0.4925 ***	-0.1869	16,814	0.1184	73,688	73,835
40	HU	0.0337	0.7178 ***	21.9173	-0.0777 *	-0.044	11,643	0.0672	52,314	52,453
41	NL	0.2054 ***	1.0254 ***	18.5694	-0.2152 ***	-0.0098	15,290	0.0813	61,375	61,520
42	NO	0.1638 ***	-0.2535	-4.0084	0.0730 *	0.2368	13,626	0.1381	56,987	57,130
43	PL	0.0097	-0.8834 ***	-21.4614	0.1299 ***	0.1396	11,946	0.0566	53,581	53,722
44	PT	0.3486 ***	1.0016 ***	27.2526	-0.2275 ***	0.1211	11,286	0.1095	49,982	50,121
45	SE	0.3080 ***	2.3734 ***	52.8965	-0.4894 ***	-0.1814	14,433	0.0997	61,792	61,936
46	SI	0.0051	1.9532 ***	50.3956	-0.2584 ***	-0.2533	8639	0.0777	38,731	38,866

Asterisks denotes statistical significance levels at *p*-values *p < 0.05, **p < 0.01 and ***p < 0.001

**Table 6:** Satisfaction with education per country

Model	Country	b1: slope 02 – 08	b2: diff 08 –10	b2: % change 08 – 10	b3: diff in slopes (10 – 18) – (02 –08)	b1 + b3: slope 10 –18	N	R -squared	aic	bic
47	BE	0.1289 ***	0.9715 ***	16.4008	-0.1909 ***	-0.0620	14,158	0.0672	58,490	58,633
48	CH	0.1156 ***	0.3553 *	5.5284	-0.0289	0.0867	12,922	0.0612	53,610	53,752
49	DE	-0.0357	-0.2749 *	-5.5273	0.1519 ***	0.1162	22,195	0.0832	96,774	96,926
50	ES	0.0801 **	-0.2314	-4.6366	-0.0602	0.0199	13,205	0.0559	57,611	57,753
51	FI	-0.0345 *	0.3265 ***	4.2558	-0.0193	-0.0538	16,282	0.0617	56,447	56,594
52	FR	0.03	0.075	1.5200	-0.0342	-0.0042	15,150	0.0386	65,245	65,390
53	GB	0.1561 ***	1.1306 ***	22.5308	-0.2240 ***	-0.0679	16,336	0.0657	69,082	69,229
54	HU	-0.2219 ***	-0.2902	-5.8700	0.2121 ***	-0.0098	10,728	0.0594	47,601	47,739
55	NL	0.0344	-0.149	-2.4565	0.0712 **	0.1056	14,544	0.0574	55,475	55,619
56	NO	0.0515 *	-0.4455 ***	-6.4946	0.1259 ***	0.1774	13,515	0.0826	53,439	53,582
57	PL	0.1947 ***	0.4404 **	8.5134	-0.1297 ***	0.0650	11,562	0.0801	51,093	51,233
58	PT	0.1802 ***	0.4207 *	10.1363	-0.0603	0.1199	10,940	0.0760	46,466	46,604
59	SE	0.1514 ***	0.7321 ***	14.4499	-0.2003 ***	-0.0489	14,077	0.0523	59,436	59,580
60	SI	0.0135	0.1048	1.9852	-0.015	-0.0015	8302	0.0445	36,467	36,601

Asterisks denotes statistical significance levels at *p*-values *p < 0.05, **p < 0.01 and ***p < 0.001

### Immediate/short-term effects

To compare the immediate effect of the crisis on satisfaction I first divided the results in Tables 3, 4, 5 and 6 based on whether b_2_ was statistically non-significant (no effect) or significant (effect). I then ranked both groups based on the b_2_ value, from the smallest to the largest. This way I could classify those countries where the crisis had had no immediate effect, those in which the effect was negative and those where the crisis had increased levels of satisfaction, all in order of magnitude.

**Fig. 1:**
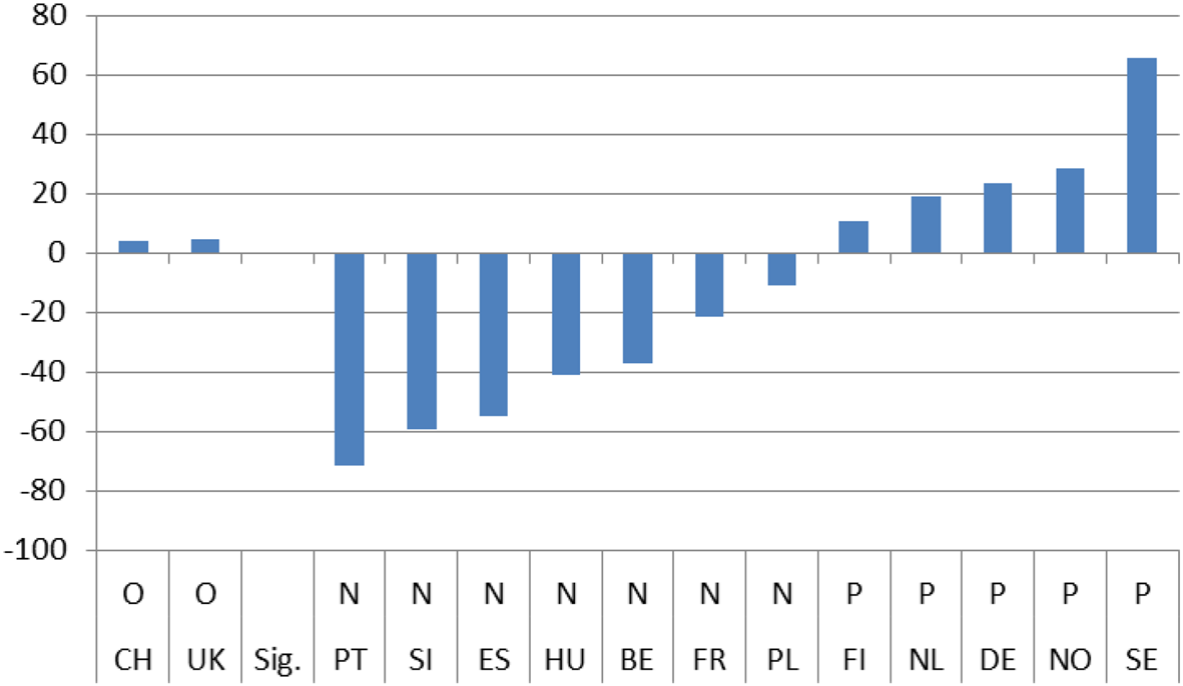
(STE GOV). Immediate Effects of crisis on satisfaction with the government (elasticities)

**Fig. 2:**
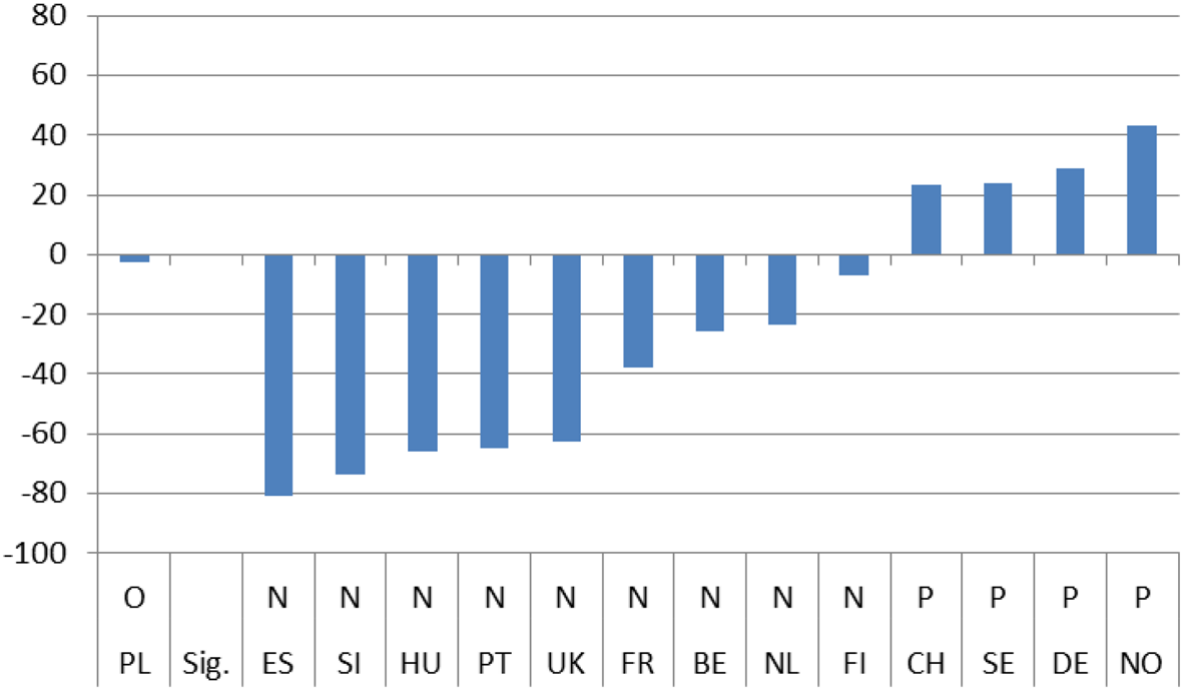
(STE ECO). Immediate Effects of crisis on satisfaction with the economy (elasticities)

**Fig. 3:**
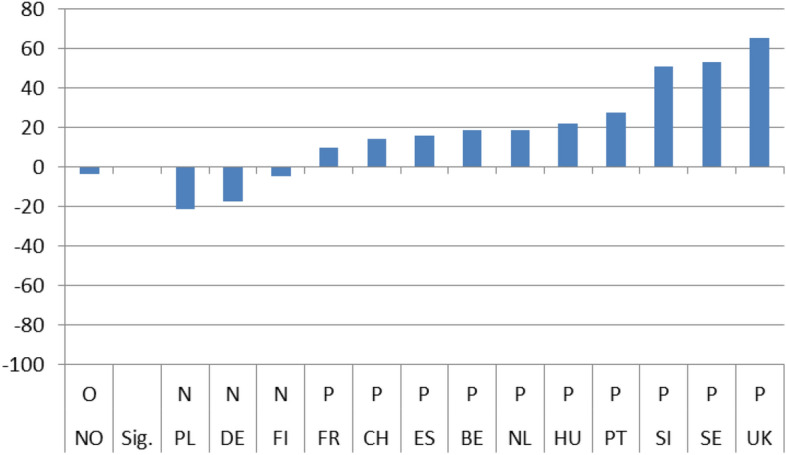
(STE HLT). Immediate Effects of crisis on satisfaction with health services (elasticities)

**Fig. 4:**
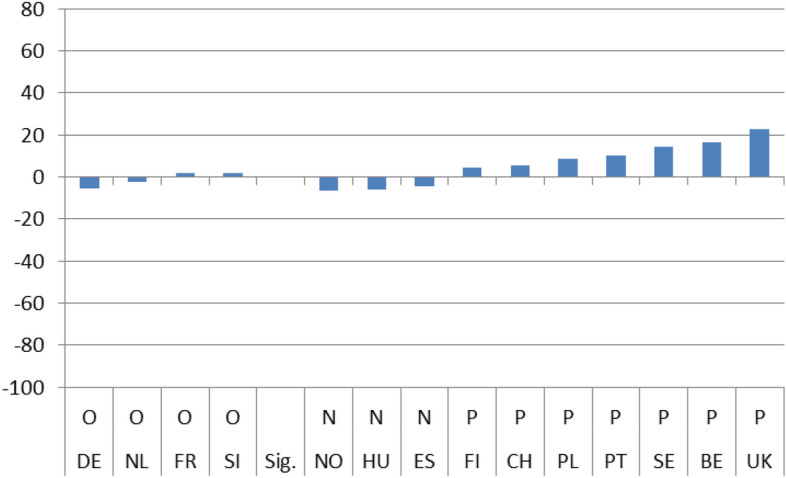
(STE EDU). Immediate Effects of crisis on satisfaction with education (elasticities)

Figures 1–4 show these country rankings. I ranked them based not on the absolute value of the potential effect (b_2_), but on the percentage change (elasticity) to account for the variability in the levels of satisfaction amongst the 14 countries in 2008.^15^ Note also that, for all three coefficients, I use the same scale on the Y axis so that the effect is comparable not only between countries but also between satisfaction types.

The immediate effects of the crisis on the four indicators are evident. In only 8 instances out of the potential 56,^16^ do we get a non-significant result for b_2_ and for its respective elasticity. As far concerns for the significant effect go, the results are mixed. There are more negative responses in relation to satisfaction with the government in general and the economy in particular. The Portuguese and Spaniards were the most dissatisfied, with the Slovenians coming closely second. On the positive side, the Swedes and the Norwegians were the most satisfied, both with their government’s operations and with its handling of the economy during the first two years after the crisis. The Germans were also quite content (Figs. 1 and 2).

Regarding satisfaction with health services and educational policies, governments seemed to have performed better immediately after the crisis. In most countries respondents’ satisfaction jumped higher in 2010 compared to 2008. The highest growth in satisfaction was observed in the UK, although for education the growth was more moderate (Figs. 3 and 4).

### Long-term/dynamic effects

**Fig. 5:**
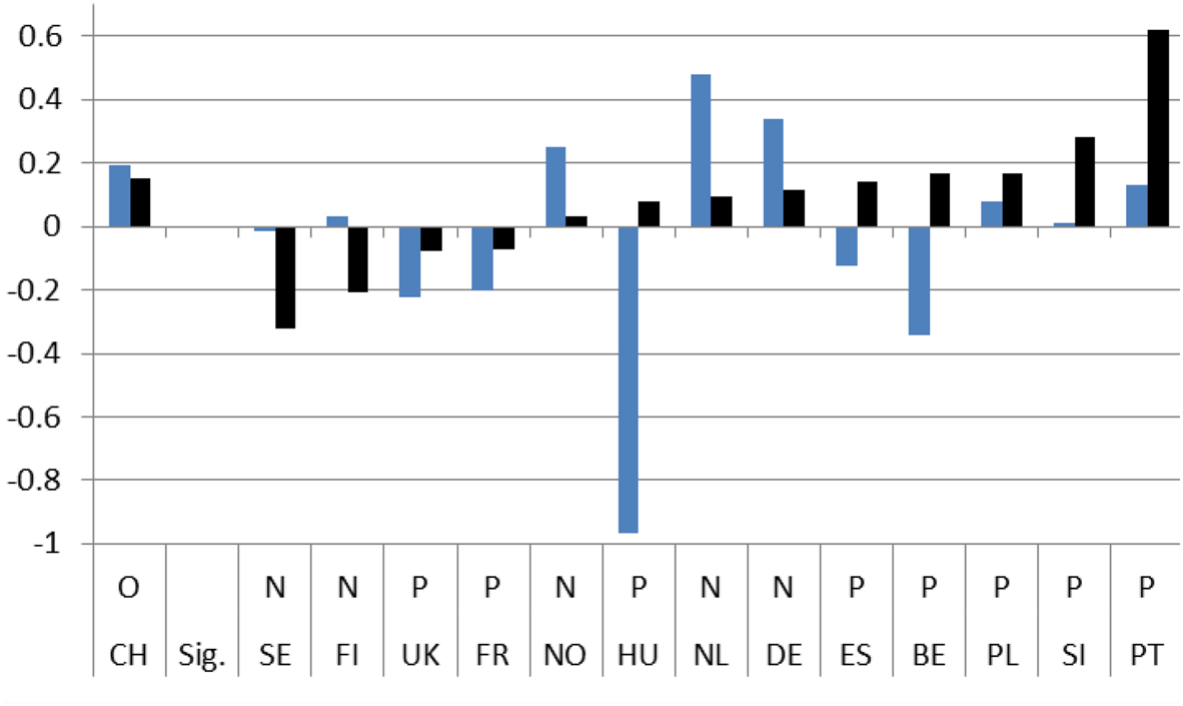
(LTE GOV). Long-term evaluation of crisis on satisfaction with the government

**Fig. 6:**
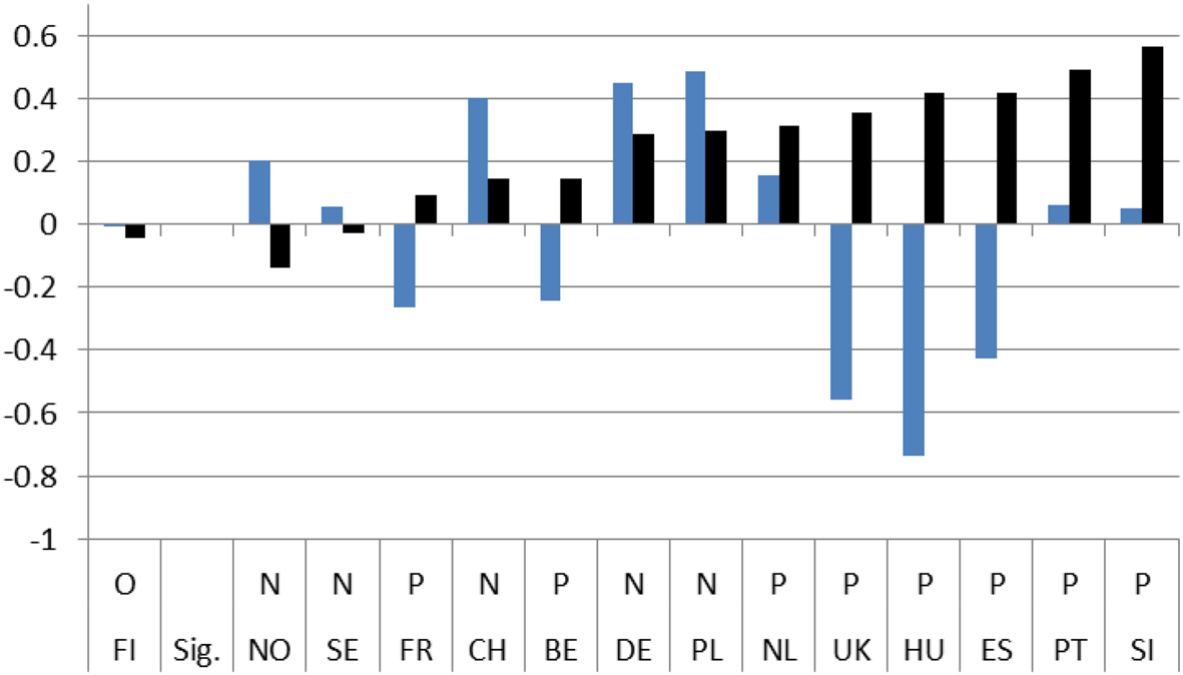
(LTE ECO). Long-term evaluation of crisis on satisfaction with the economy

**Fig. 7:**
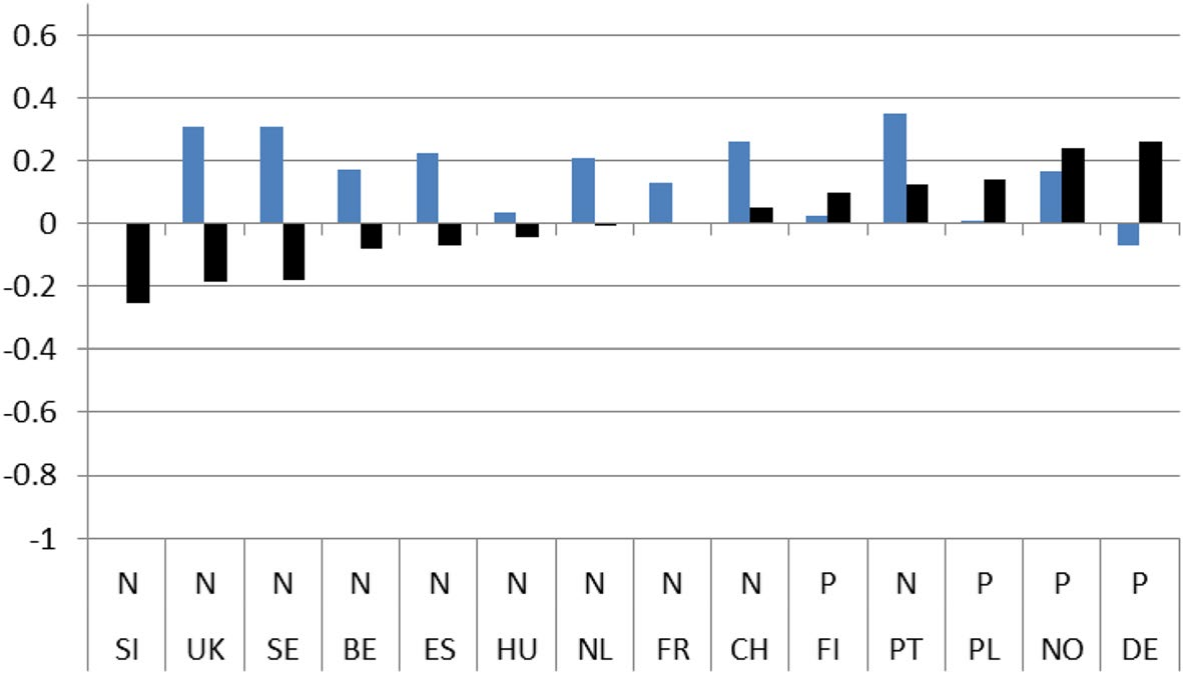
(LTE HLT). Long-term evaluation of crisis on satisfaction with health services

**Fig. 8:**
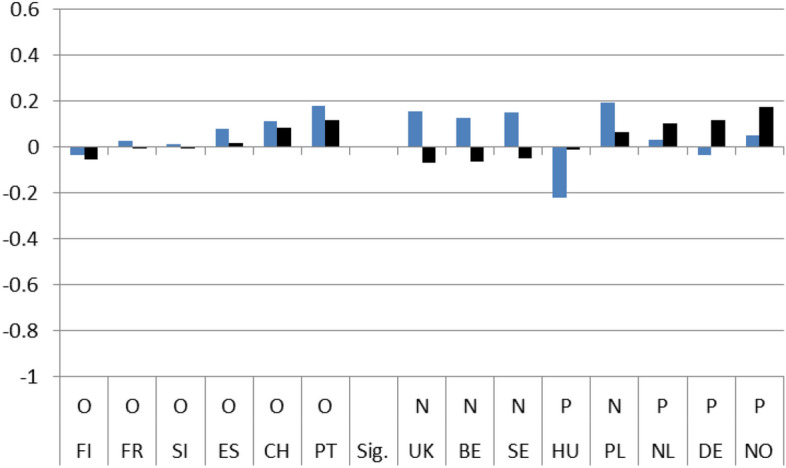
(LTE EDU). Long-term evaluation of crisis on satisfaction with education

The next 4 Figures show the long-term dynamic effect of the crisis on the 4 indicators of interest. As with Figs. 1 to 4, Figs. 5, 6, 7 and 8 use the same scale on the Y-axis and have been divided into two groups based on whether the differences (b_3_) are statistically significant (those to the right) or not (left). Within the two groups, they have then been ranked based on the magnitude of the slope after the crisis (b_1_ + b_3_) per country, from the smallest to the largest (dark column). To capture the dynamic effect of the crisis I depict the growth in satisfaction both before (b_1_-light column) and after the crisis (b_1_ + b_3_ –dark column). The columns are shown next to each other to emphasize the difference in growth from one period to the other.

Overall, after the crisis, satisfaction seems to have grown positively for the majority of cases. The slope of satisfaction with the government comes out positive in 9 countries and with the economy in 11. With health services and education, positive growth is reported in fewer countries (7 and 4 respectively) and, in addition, the magnitude of growth is much smaller. On aggregate, we have in 8 instances out of 56 (14,28%) similar (non-significant) growth before and after the crisis, in 31 instances (55,36%) statistically significant positive growth and in 17 (30,3%) statistically significant negative growth.

By individually ranking the countries we can see that after the 2008 crisis, satisfaction growth with the government was found to be highest in Portugal and lowest in Sweden, with the economy it was highest in Slovenia and lowest in Norway, with health services it was highest in Germany and lowest in Slovenia, and finally, with educational services, the highest satisfaction growth was recorded in Norway and the lowest in the UK.

In addition for each country, a line under the X axis evaluates the overall impact of the exogenous 2008 shock. If the country belongs to the non-significant difference group (on the left), there is no effect observed (O). If the difference between the pre-and post-crisis period slopes is statistically significant, the evaluation of the effect can be Negative (N) or Positive (P) based on the sign of the b_3_ coefficient. This means that even if the sign of the slope after the crisis is positive the evaluation can still be negative. This is the case with 4 countries (UK, FR, NL, DE) in Fig. 5 depicting satisfaction with the government, with three countries (CH, DE, PL) in Fig. 6 depicting satisfaction with the economy, with three countries (FR, CH, PT) in Fig. 7 depicting satisfaction with health services, and with one country ( PL) in Fig. 8 depicting satisfaction with education.

Hence, although the slope of satisfaction is mostly positive after the 2008 crisis overall, the net effect seems to be slightly less favorable. For the government a positive effect (P) is reported in 8 countries and the same goes for the economy. On the other hand, for health services as well as for education a positive effect (P) is found only in 4 countries.

## Summary and discussion

In this study I examined the individual level of satisfaction in 14 European countries, for a 16-year period, from 2002 to 2018. The data analyzed was gathered from the European Social Survey. The indicators of satisfaction were linked to the performance of government overall and in relation to three specific policy areas; the economy, health services and education. During the period under scrutiny the exogenous shock generated by the global financial crisis of 2008 compelled many governments to implement different fiscal, monetary and reform policies to combat the severe recession that followed. With the available time series data, I measured how the crisis was associated with these indicators of satisfaction, both short-and long-term.

The motivation for the analysis is twofold. First it is due to the COVID-19 global crisis which resembles somewhat the events of 2008, but on a much wider and severer scale. Governments are now under immense pressure to solve several problems, such as economic, health and education-related without delays. The losses in human life have easily surpassed those in many battle fields of the twentieth century and that, in a much shorter time span. Businesses- especially small and medium sized (SMEs) - have closed or are in the brink of closing, while unemployment has risen sharply. Schools and universities have had to lock their facilities while distance teaching has been enforced on a wide scale.

Governments and public sector officials are constantly being evaluated on how they are handling the unprecedented calamity generated by this exogenous shock. Thus, examining not only the overall performance of governments during the 2008 crisis but also how they fared particularly in these three vital policy areas - the economy, health services and education- may provide us with a baseline to match and compare with the respective levels generated by the current COVID-19 crisis.

Second, at a more theoretical level, policies influence people’s wellbeing and behavior. Economic policies in particular play a vital role, especially in the light of exogenous events (Gahner-Larsen et al. 2019, p. 234). Governments need feedback mechanisms (e.g. through satisfaction surveys) from citizens on their merits and faults. This in turn influences future policy planning and implementation. Hence and irrespective of the COVID-19 or the 2008 crisis, examining how governments perform is of academic interest in itself.

On aggregate, the results indicate that during the period immediately after the crisis, from 2008 up until 2010, individual satisfaction with governments in general and the economy in particular, dropped considerably; for health and education related policies satisfaction grew. After the crisis, the slopes of all indicators came out statistically significant with a positive sign. Comparing the pre-and post-crisis slopes, satisfaction with the government in general and with the economy in particular grew faster after the crisis. The opposite was the case for health services and education policies. Their slopes grew as well, but at a pace slower than before the crisis. Applying the same analysis for each country separately, the results were mixed. There was no indication of any country with consistently low or high coefficient values for any of the four satisfaction indicators. In the four pairs of Figures for each type of satisfaction (Figs. 1 and 5, 2 and 6, 3 and 7, 4 and 8) we see interesting behavior patterns within each country. In the majority of cases (20 + 23 = 43 out of 56) if the short-term effect (STE) is negative (N), the long-term effect (LTE) is positive (P) and vice-versa. Table 7 depicts these effects for each country and each type of measured satisfaction. Overall, short- and long-term effects of the 2008 crisis seem to have balanced out. The switch from short-term negative to long-term positive satisfaction could be interpreted as reflecting the successful efforts of governments to correct the immediate dissatisfaction felt by many due to the 2008 crisis. In contrast, the long-term reduction in satisfaction compared to the short-term positivity is not as easily explained. It could be that some individuals seeing the problems other countries faced applauded their own government’s short-term performance in handling the crisis. Long-term however, they became more critical, both overall and regarding the three individual policies under scrutiny.

**Table 7:** Short (STE) and long-term (LTE) effects of the 2008 crisis on satisfaction

**Satisfaction**	**GOV STE**	**GOV LTE**	**ECO STE**	**ECO LTE**	**HLT LTE**	**HLT STE**	**EDU LTE**	**EDU LTE**	
**Country**									
**BE**	N	**P**	N	P	P	N	P	N	
**CH**	O	O	P	N	P	N	P	O	
**DE**	P	N	P	N	N	P	O	P	
**ES**	N	P	N	P	P	N	N	O	
**FI**	P	N	N	O	N	P	P	O	
**FR**	N	P	N	P	P	N	O	O	
**UK**	O	P	N	P	P	N	P	N	
**HU**	N	P	N	P	P	N	N	P	
**NL**	P	N	N	P	P	N	O	P	
**NO**	P	N	P	N	O	P	N	P	
**PL**	N	P	O	N	N	P	P	N	
**PT**	N	P	N	P	P	N	P	O	
**SE**	P	N	P	N	P	N	P	N	
**SI**	N	P	N	P	P	N	O	O	
**STE-LTE**									**Totals**
**N-P**		7		8		3		2	20
**N-O**				1				1	2
**N-N**									
**P-N**		5		4		10		4	23
**P-O**								3	3
**P-P**									
**O-P**		1				1		2	4
**O-N**				1					1
**O-O**		1						2	3
**Totals**		14		14		14		14	56

*P*: Positive effect; *N*: Negative effect; *O*: No effect, *GOV*: Government; *ECO*: Economy; *HTL*: Health; *EDU*: Education

There are some caveats in the study. They stem mainly from the limitation of the data utilized and the implemented analytical method. First, external validity is not achieved. We cannot generalize the findings because of the low number of countries in the data. Nonetheless, by choosing only those countries that participated in all the survey rounds, I created a longer time series cross-sectional data set, and thus was able to run the ITS regression models. Furthermore, the ITS design requires at least 8 observational instances for the two periods, before and after the exogenous intervention. Since in my dataset I had only 9 in total (4 + 5), I bootstrapped all the models to ensure that the confidence intervals of the variable coefficients were of the correct size. Consequently this made the statistical significance of the coefficients more robust. Lastly, there is the school of thought which argues that, regardless of survey results, people respond to their evaluation of governments based not on facts, but on pre-existing beliefs (Hvidman 2019, p. 265). I did not test this assertion. This may indeed be a topic for future research wherein one combines evaluative responses on specific government policies with general perceptions on the functions and ethics of public sector officials and politicians.

At the beginning of August 2020 there were almost 20 million people in 213 countries that were infected with the virus; approximately 725 thousand had perished. In less than four months later, by the end of November 2020 the respective number of infections globally had increased threefold to 60 million, whereas the number of deaths had almost doubled, to a shocking 1.4 million. In the 14 countries I examine in this study, there were more than 9.3 million people infected and 225 thousand dead. The lockdown is going to shrink global economic growth dramatically, with the GDP in many countries estimated to drop by an average of 6% to 8% during 2020 (IMF, 2020, p.v). In its latest report, the European Commission (2020) forecasts that the European economy in particular will contract by an average of 8.75% in 2020, before recovering at an annual growth rate of 6% in 2021.^17^

In Table 8 the mortality rate per million inhabitants ranges between 58 in Norway to 1373 in Belgium. The same variability is observed in the GDP growth. During the summer of 2020, it ranged between -4.6% in Poland and -10.9% in Spain. In its forecasts for 2021, the European Commission predicts that all the economies of the 14 countries will recover, at different paces. The net GDP loss during the next 2 years will be-3.8% for Spain and Portugal, but only-0.3% for Poland and-0.5% for Switzerland.

**Table 8:** Reported cases, deaths in connection with COVID-19 and GDP forecasts

Country	Total Cases	Total Deaths	Deaths/1 M pop	Population	GDP % change Summer 2020	GDP % change Summer 2021	Difference 2021–2020
**BE**	561,803	15,938	1373	11,609,863	-8.8	6.5	-2.3
**CH**	304,593	4308	496	8,679,947	-5.0	4.5	-0.5
**DE**	964,909	15,007	179	83,891,567	-6.8	5.3	-1.5
**ES**	1,614,126	43,668	934	46,762,073	-10.9	7.1	-3.8
**FI**	22,652	384	69	5,544,183	-6.3	2.8	-3.5
**FR**	2,153,815	50,237	769	65,331,590	-10.6	7.6	-3.0
**UK**	1,538,794	55,838	821	68,028,987	-9.7	6.0	-3.7
**HU**	185,687	4114	426	9,650,444	-7.0	6.0	-1.0
**NL**	493,744	9035	527	17,150,117	-6.8	4.6	-2.2
**NO**	33,717	314	58	5,438,194	-5.5	3.0	-2.5
**PL**	924,422	14,988	396	37,829,891	-4.6	4.3	-0.3
**PT**	268,721	4056	398	10,184,697	-9.8	6.0	-3.8
**SE**	225,560	6500	642	10,124,489	-5.3	3.1	-2.2
**SI**	69,306	1199	577	2,079,053	-7.0	6.1	-0.9
**Totals**	9,361,849	225,586					

Sources: www.worldometers.info/coronavirus, Accessed Nov 25, 2020; European Commission (2020)

Based on these indicators we can safely assume that that peoples’ satisfaction levels will vary from country to country, both during and after the coronavirus pandemic. In countries such as Finland and Norway, where the death toll per million is relatively low, satisfaction with health services could end up being much higher compared for example to Belgium, Spain, the UK or France. Further, satisfaction with the economy will most probably be inversely correlated to the magnitude of the net GDP loss during 2020 and 2021. This is because reduction in GDP usually means revenue losses, bankruptcies, higher unemployment and fewer investments. In addition, both the COVID-19 number of victims and the GDP figures may also affect satisfaction with the government, in general. Finally, when it comes to the satisfaction with education, it is difficult to evaluate in advance how it will be affected by the COVID-19 pandemic. It  will perhaps depend on the operational instructions given by each country’s ministry of education, on the technological infrastructure already in place for distance learning in each country, on whether employers encourage work from home or other arrangements.

The European Social Survey announced that during the fall of 2020 it will begin conducting its 10^th^ round of surveys with at least 26 countries participating. Although we cannot have a complete time series data for which we could examine past trends in their behavior, a simple pre-and post-crisis data set with responses from the 2018 and the 2020 surveys will be available from most of the participating countries. This will provide us with sufficient information to measure the short term effects of the pandemic on the topics investigated in this study so that a comparison of results can be made.^18^

## Data Availability

All data and code for all regression models (in Stata 14.2) and Figures (Excel) in the manuscript are available upon request.

## Footnotes

1 https://www.bbc.com/news/business-52113841.

2 https://www.bbc.com/news/business-52350082.

3 For example, on June 4, 2020, the European Central Bank (ECB) announced among other measures that “…the pandemic emergency purchase program (PEPP) will be increased by 600 billion to a total of 1,350 billion euro”. (https://www.ecb.europa.eu/press/pr/date/2020/html/ecb.mp200604~a307d3429c.en.html)

4 Additional borrowing from the financial markets increases a country’s sovereign dept. As discussed above due to the crisis and the lockdown, the GDP will decrease. Hence the debt to GDP ratio will become even higher.

5 The financial crisis of 2008 and the COVID-19 are indeed exogenous events which have produced negative shocks to the economy and other sectors of governmental activity globally. In the strict sense of the word, both these crises are *not* exogenous in the 2 countries in which originated, the United States and China respectively.

6 BE-Belgium, CH-Switzerland, DE-Germany, ES- Spain, FI- Finland, FR-France, UK-United Kingdom, HU-Hungary, NL-Netherlands, NO-Norway, PL-Poland, PT-Portugal, SE- Sweden, SI-Slovenia. Although Ireland has participated in all nine ESS rounds, it was dropped from the list for non-compatibility reasons. In the 2002 survey (round 1) instead of being asked “How satisfied are you with your government”, the Irish were asked “How satisfied are you with your Parliament”.

7 Fixed effects in relation to the country dummies.

8 Shadish et al. (2002, p.108) calls it the One-Group Pretest–Posttest Design.

9 Shadish et al. call it Simple Interrupted Series Design.

10 The possibility of non-linear growth (e.g. quadratic) of satisfaction, pre and/or post the crisis, is not examined here due to the small number of observations. But it is indeed an interesting hypothesis.

11 This is the basic logic behind the approach. As I shall describe later, the actual evaluation of the results is based on interpreting the value, the sign and the statistical significance of more than one coefficient.

12 I have applied bootstrapping (400 repetitions) in all ITS models reported in the paper. Additionally, instead of the standard 95%, a 99.99% level is used in all models when calculating confidence intervals. Therefore the statistical significance reported, is on the conservative side.

13 See also footnote 10 regarding non-linearity.

14 The coefficients of the individual level control variables for each model are not shown but are available upon request.

15 That is, the absolute change from 2008 to 2010 would not have been comparable if the mean satisfaction value in 2008 were not similar in all countries. For example, if the average satisfaction in 2008 is three and in 2010 four, the difference of one depicted by b_2_ represents a 33% increase; if in 2008 the average satisfaction is five, a positive difference of one from 2008 to 2010 represents a 20% increase.

16 56 = 14 countries × 4 satisfaction indicators.

17 www.worldometers.info/coronavirus. Accessed August 8, 2020 and November 25, 2020.

18 https://www.europeansocialsurvey.org/about/singlenew.html?a=/about/news/essnews0083.html. Accessed July 10, 2020.

## Code availability

As above.
